# Efficacy and safety of heparin for sepsis-induced disseminated intravascular coagulation (HepSIC): study protocol for a multicenter randomized controlled trial

**DOI:** 10.1186/s13063-023-07853-5

**Published:** 2024-01-02

**Authors:** Yini Sun, Renyu Ding, Hao Sun, Yingjian Liang, Xiaochun Ma

**Affiliations:** 1 Department of Critical Care Medicine, The First Affiliated Hospital of China Medical University, Shenyang, China; 2 Department of Clinical Epidemiology and Evidence-based Medicine, The First Affiliated Hospital of China Medical University, Shenyang, China

**Keywords:** Sepsis, Disseminated intravascular coagulation, Heparin, Randomized controlled trial

## Abstract

**Background:**

Disseminated intravascular coagulation (DIC) occurs in 30–50% of septic patients and contributes to high mortality in the intensive care unit (ICU). However, there are few proven interventions for coagulation disorder management in sepsis. Experimental and clinical data have demonstrated that sepsis could benefit from unfractionated heparin (UFH) treatment. To date, there are no large multicenter trials to determine the safety and efficacy of UFH in septic patients with suspected DIC.

**Methods:**

A multicenter, double-blinded, placebo-controlled randomized trial is designed to recruit 600 patients who met sepsis 3.0 criteria and suspected DIC. Participants will be randomized (1:1) to receive UFH or saline via continuous intravenous administration for 7 days within 6 h of enrolment. The primary outcome is ICU mortality. The secondary outcome includes 28-day all-cause mortality, the improvement of Sequential Organ Failure Assessment scores, and the incidence of major hemorrhage. Investigators, participants, and statisticians will be blinded to the allocation.

**Discussion:**

The HepSIC trial is to evaluate the efficacy and safety of UFH on sepsis-related DIC across different areas of China. The small dosage of UFH administration would offer a new potential approach for treating sepsis-related coagulation disorders.

**Ethics and dissemination:**

Ethical approval was granted by all the ethics committees of 20 participant centers. Results will be disseminated via peer-reviewed publications and presented at conferences.

**Trial registration:**

ClinicalTrials.gov NCT02654561. Registered on 13 January 2016.

**Supplementary Information:**

The online version contains supplementary material available at 10.1186/s13063-023-07853-5.

## Background

Sepsis is a life-threatening organ dysfunction caused by dysregulated host responses [1]. Over 48 million people become septic globally each year, with 11 million dying from the disease, representing nearly 20% of all deaths in the world [2]. A total of 30–50% of septic patients suffer from overt disseminated intravascular coagulation (DIC), which contributes to increased mortality in these patients [3, 4].

The release of proinflammatory factors promotes the activation of the coagulation system. Coagulation activation and endothelial injury contribute to clot formation [5, 6]. In turn, the procoagulant status amplifies the inflammatory response, leading to adverse outcomes of sepsis. Of note, sepsis induces the loss of some crucial anticoagulants, such as protein C, antithrombin (AT), and thrombomodulin (TM) [7, 8]. Therefore, the rationale of some anticoagulant therapy is to replenish these downregulated anticoagulants. Clinical evidence about the efficacy of anticoagulation therapy in sepsis remains insufficient. Although recombinant human activated protein C (rhAPC) has been withdrawn from the market, the PROWESS study demonstrated that rhAPC showed a survival benefit in severely septic patients, especially in a subpopulation of overt DIC patients [9]. However, the KyberSept trial demonstrated that high-dose AT III administration had no survival benefit with an increased risk of hemorrhage in severe sepsis or septic shock patients [10]. A randomized controlled trial showed that recombinant thrombomodulin (rhTM) had an increasing trend in survival in sepsis with suspected sepsis-associated DIC [11]. Recently, a meta-analysis of randomized controlled trials suggested that the survival benefit of anticoagulants was observed only in the sepsis with DIC population [12]. Although the SCARLET trial did not show a survival benefit of rhTM among patients with sepsis-associated coagulopathy [13], a post hoc analysis demonstrated that patients with higher thrombin generation markers had lower mortality with the administration of rhTM [14]. Therefore, septic patients with coagulation disorders could benefit from suitable anticoagulation therapy.

However, these replenishment treatments are only suitable for specific subpopulations, and the expense of these medicines is dramatically too high to afford for middle-low income countries. In contrast, unfractionated heparin (UFH), which is less than 1 dollar per ampoule, does not simply supplement what sepsis patients have depleted. Emerging evidence suggests that UFH exerts anti-inflammatory and immunomodulatory properties and protects the endothelial glycocalyx from shedding in addition to its well-known anticoagulant effects [15–17]. Several clinical studies and meta-analyses have demonstrated that heparin may decrease 28-day mortality, while reports on safety outcomes, including the occurrence of bleeding, are insufficient [18–20]. Jaimes et al. reported no survival benefit of UFH in a single-center randomized clinical trial. However, the patients in this study were suspected of sepsis and not patients with coagulation disorder [21]. Additionally, these clinical trials were conducted with small sample sizes in a single center, limiting the evidence of potential benefit in patients with more severe sepsis with coagulopathy.

Therefore, more robust clinical evidence is warranted to evaluate the efficacy and safety of UFH in septic patients with coagulation disorders. Here, we designed a multicenter, randomized controlled trial (RCT) to determine whether the early administration of UFH is efficacious and safe in the treatment of septic patients with suspected DIC in the ICU.

## Methods and trial design

### Study design

**Fig. 1:**
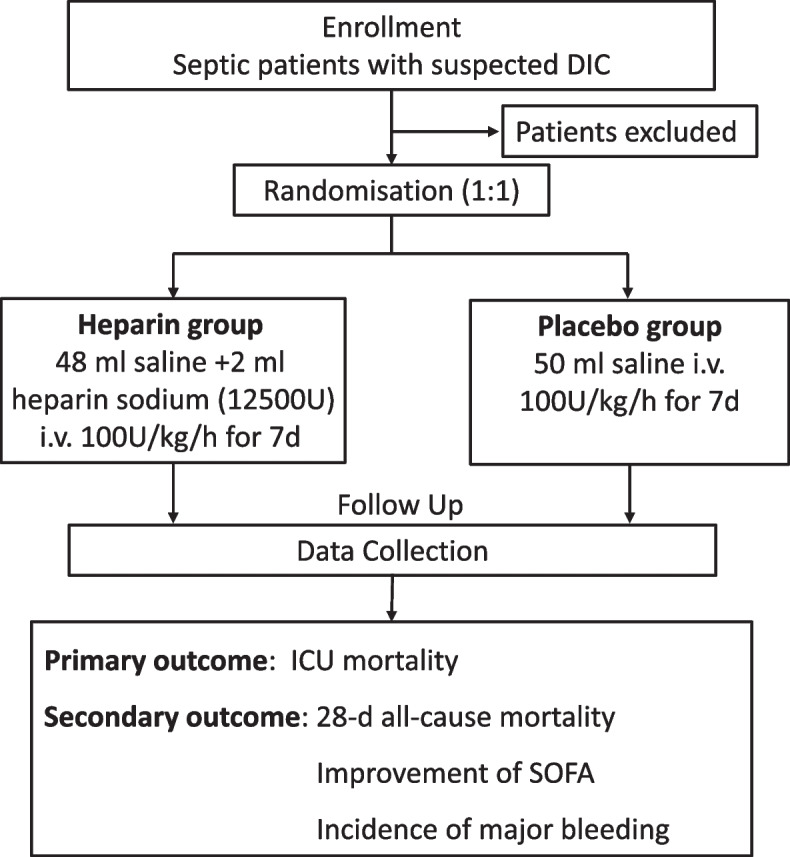
The flow chart of the HepSIC study. DIC, disseminated intravascular coagulation. ICU, intensive care unit; SOFA, Sequential Organ Failure Assessment

The HepSIC study is a multicenter, double-blind, randomized, placebo-controlled trial. The study protocol was approved by the Research and Ethics Committee of the First Affiliated Hospital of China Medical University ([2015]2015-113-2, Shenyang, China). Participants will be allocated randomly to two arms at a 1:1 ratio: the heparin group and the control group. The control group will receive saline only, while the heparin group will receive continuous intravenous UFH administration within 6 h of enrollment for 7 consecutive days. Blinding will be maintained for researchers, participants, and statisticians during the entire study. The study protocol is summarized in a flow chart (Fig. 1). The study protocol follows the Standard Protocol Items: Recommendations for Interventional Trials statement recommendations (Supplemental Fig. 1).

### Study participants

Participant enrollment is currently ongoing from all the ICUs of 20 hospitals in China. The 20 ICUs are from 20 university hospitals integrating clinical care, scientific research, and teaching across 11 provinces in Northeast China, Northwest China, Southwest China, and East China (see Supplemental Fig. 2 for the participating hospitals). Patients who meet the inclusion criteria and no exclusion criteria at randomization and sign the informed consent forms will be enrolled in the study. The consent form includes the collection and use of participant data and biological specimens. Enrolment must be completed within 12 h after patients are admitted to the ICU. The study period is planned from April 2018 to December 2024.

### Inclusion criteria

Participants will simultaneously meet the following criteria:

1. Patients aged ≥18
2. Diagnosed as sepsis according to the criteria of sepsis 3.0 (The Third International Consensus Definition for Sepsis and Septic Shock) [1]
3. Suspected DIC was assessed with modified ISTH criteria based on platelet count, prothrombin time, and fibrin/fibrinogen degradation products (Table 1).

**Table 1:** Modified disseminated intravascular coagulation algorithm

Variable (units)	Value	Points
Platelet count (× 10^9^/L)	≥100	0
	50–99 or >30% decrease within 24 h	1
	<50 or >50% decrease within 24 h	2
Prothrombin time prolongation (s)	<3	0
	3–6	1
	>6	2
D-dimer (μg/mL)	<2	0
	2–8	2
	>8	3
Total needed for study entry		≥4

### Exclusion criteria

Patients will be excluded if they fulfill any of the exclusion criteria.

1. Pregnant or lactating women.
2. Patients with a history of allergy to heparin.
3. Significant hemorrhage or high risk of bleeding, including
  In the acute phase of trauma or active bleeding
  History of severe brain injury, intracranial surgery or stroke, cerebral aneurysm, arteriovenous malformation within 3 months
  A history of congenital hemorrhage (e.g., hemophilia)
  Underlying fulminant hepatitis, decompensated cirrhosis, or other severe liver diseases
4. Use of the following medicine:
  Heparin and heparin analogs (e.g., low molecular weight heparin, dalteparin, tinzaparin) within 12 h before treatment
  Warfarin treatment within 7 days and abnormal INR value
  Thrombolytics within 72 h prior to study dosing
  Other anticoagulant drugs, including factor Xa inhibitors (e.g., apixaban, rivaroxaban, edoxaban) and direct inhibitor IIa (e.g., dabigatran)
5. Patients with an expected ICU stay of less than 48 h.
6. Cardiopulmonary resuscitation within 7 days prior to enrollment.
7. Participating in other clinical trials in the previous 30 days.
8. Irreversible diseases, such as the late stage of malignant tumors or the terminal stage of other diseases.

### Risks, AEs, and informed consent

The protocol is designed to ensure the safety of participants. On the one hand, the dose of unfractionated heparin is moderate for critically ill patients. We calculated the dose of UFH or placebo according to the body weight of each participant, and UFH was administered continuously intravenously at 100 U/kg/24 h (no more than 12,500 U per 24 h). On the other hand, patients with greater potential risk were excluded based on exclusion criteria. Of note, adverse events (AEs) and serious adverse events (SAEs) will be observed and recorded during the 28 days of study from enrollment in accordance with the practice guidelines issued by the National Medical Products Administration of the People’s Republic of China. Investigators will evaluate the relationship between events and the intervention and report the AEs and SAEs to the central ethics committee and the data and safety monitoring board.

### Recruitment

All potential participants are approached by a clinician and a researcher to assess eligibility, provide research information, and obtain informed consent. Participants will be informed of the study purpose, procedures, benefits, and risks and will be given adequate time to consult and consider participation. If the participants cannot sign the consent form, a legally authorized representative will do it on their behalf. The consent process will comply with the guidelines outlined in the Helsinki Declaration and the Good Clinical Practice International Coordinating Committee.

Our research team will manage and monitor the trial progress of the 20 participating centers through our created website, which ensures adequate participant enrolment. During the study period, the eligible patients will be enrolled based on predetermined inclusion and exclusion criteria. The screening logs will be maintained on the website to document reasons for excluding candidates.

### Randomization and allocation concealment

There are 20 participant ICUs with 20 investigators. The eligible participants who meet the inclusion criteria and provide informed consent will be randomized using a software-generated randomization number. Each ICU is assigned an independent, random numeric table by the center primary investigator. There are equal numbers of 1 and 2, which represent the control group (sterile saline) and the heparin group (UFH), respectively. Once an eligible participant has enrolled, the independent drug administrators will obtain group information from the assigned table according to the chronological order of recruitment and assign the study drugs to the nurses for administration.

### Interventions

The participants in the heparin group will receive unfractionated heparin (0.9% saline 48 ml + heparin 2 ml) via continuous intravenous infusion for 7 days. The total dose for a 24-h period was less than 12,500 international units (1 ampule) in the heparin group. The heparin ampules (12,500 U per 2 ml) were manufactured by Shanghai No. 1 Biochemistry Company. The other participants in the placebo group will only receive 50 ml of 0.9% saline via intravenous administration. The method and speed of infusion were identical to those of the heparin group. The study drugs will be commenced within 6 h of enrollment for a 7-day period. Participants in the two groups will receive standard care from the attending physician according to the International Guidelines for Management of Sepsis and Septic Shock [1]. Ulinastatin, Xuebijing, and other anticoagulation drugs were not allowed during the study period. Regional citrate anticoagulation is strongly recommended for continuous renal replacement treatment.

### Blinding

An independent drug administrator (commonly a clinical research nurse) in each center will be in charge of preparing the placebo or heparin. The 50 ml transparent syringes were used for drug administration for both groups to ensure an identical appearance. Procedures for administration are identical and will be performed by the nurses in charge of each patient’s treatment. The independent drug administrator will receive group information based on the random numeric table when the available participant enrolls. Once the assignment is known, the drug administrator—who is the only person for whom the link between the sequential ID and the drug is available—instructed a research assistant to adhere the label with the ID to the appropriate syringe containing UFH or saline. The participants, investigators, clinical doctors, and nurses will be blinded to the study drug assignment except for the drug administrator.

### Criteria for exiting or suspending the trial

1. Infusion is to be stopped if there is major or life-threatening bleeding, such as intracranial bleeding, gastrointestinal hemorrhage, or severe nosebleeds. If there are some signs of mild bleeding, such as coffee-like stomach contents, mild gum, or skin mucosa bleeding, the physician should closely monitor and judge according to the actual situation.
2. Infusion was suspended 2 h before major surgery and resumed 12 h after the surgery.
3. If the patient at high risk for thrombosis events, such as venous thromboembolism or acute myocardial infarction, must be treated with anticoagulation therapy, the trial needs to be stopped.
4. If there is an unexplained decline of 50% or greater in platelet counts, especially onset between day 5 and 10 after treatment, or the patient meets the 4Ts scoring system for heparin-induced thrombocytopenia (HIT) diagnosis [22], or a positive test of platelet serotonin-release assay (SRA)

### Outcomes

The primary outcome of the study is ICU mortality. The secondary outcomes include the following: a) 28-day all-cause mortality; b) improvement in SOFA scores; c) improvement in the Japanese Association for Acute Medicine (JAAM) and International Society on Thrombosis and Hemostasis (ISTH) scores; and d) bleeding risks during the study period. The daily bleeding monitor sheet is shown in Table 2.

**Table 2:** Daily bleeding monitor sheet

Classification	Manifestation	Intervention
Major bleeding	- Intracranial bleeding - Gastrointestinal hemorrhage (haematemesis, tarry stool) - Severe nose bleeding - Other fatal bleeding	Stop infusion of study drugs and recorded in the CRF
Minor bleeding	- Mild gum bleeding - Mild epistaxis - Airway mucosa bleeding - Puncture site bleeding - Bruise or skin purpura	Attending physicians monitor and make a decision

*CRF*, case report format

### Plans to promote participant retention and complete follow-up

It is not applicable because participation by the patient is only required while in the ICU. Data will be recorded upon enrollment and be followed up for 28 days. The detailed time points are listed in Table 3. The study period includes the screening period (D0, before enrollment), the intervention period (D1–D7), discharge from the ICU, and the 28-day follow-up period. The hospitalization and 28-day outcome require the hospital electronic information system or telephone-based follow-up strategy (questioning the patients or their family members over the phone). If the participants discontinue or deviate from intervention protocols, they will be encouraged to complete the follow-up of the study.

**Table 3:** Study period

Time point	Enrollment	Intervention					Follow-up	Follow-up
	D0	D1	D2	D3	D5	D7	Discharge of ICU	D28
**Enrollment**								
Informed consent	✕							
Inclusion/exclusion	✕							
Randomization	✕							
**Intervention**								
UFH		✕	✕	✕	✕	✕		
Normal saline		✕	✕	✕	✕	✕		
**Assessment**								
Demographic data	✕							
Primary disease	✕							
Base condition	✕							
Blood routine	✕	✕	✕	✕	✕	✕	✕	
Serum chemistry examinations^a^	✕	✕	✕	✕	✕	✕	✕	
Coagulation function^b^	✕	✕	✕	✕	✕	✕	✕	
Inflammation index (PCT, CRP)		✕		✕		✕		
Blood gas	✕	✕	✕	✕	✕	✕	✕	
SOFA score		✕	✕	✕	✕	✕	✕	
APACHE II score		✕	✕	✕	✕	✕	✕	
ISTH score	✕	✕	✕	✕	✕	✕	✕	
JAAM score	✕	✕	✕	✕	✕	✕	✕	
Mechanical ventilation	✕	✕	✕	✕	✕	✕	✕	✕
CRRT	✕	✕	✕	✕	✕	✕	✕	✕
Duration in ICU							✕	
Outcome							✕	✕
AEs/SAEs		✕	✕	✕	✕	✕	✕	✕

*ICU*, intensive care unit; *UFH*, unfractionated heparin; *SOFA*, Sequential Organ Failure Assessment; *APACHE II*, Acute Physiology and Chronic Health Evaluation II; *ISTH*, International Society on Thrombosis and Hemostasis; *JAAM*, Japanese Association for Acute Medicine; *CRRT*, continuous renal replacement therapy; *AEs*, adverse events; *SAE*, severe adverse events

^a^Serum chemistry includes ALT, AST, TBIL, ALB, Pre-ALB, Scr, BUN, BNP, and cytokines

^b^Coagulation indices include PT, INR, APTT, Fibrinogen, D-D, FDP, and AT-III

### Sample size calculation

The sample size calculation was based on a previous study [18]. With a significance level of 0.1, test power of 80%, mortality of severe sepsis in the control group of 42.1%, mortality in the heparin group of 31.7%, and a 12% dropout rate, a total of 600 participants (300 in each group) will be needed.

### Data collection and management

Upon enrollment, baseline data, including epidemiologic characteristics, underlying disease, main diagnosis, Acute Physiology and Chronic Health Evaluation (APACHE II), SOFA, JAAM, and ISTH scores, infection sources and pathogens, and organ function indices, will be collected. General vital signs, infection indices (white cell count, neutrophil count, lymphocyte count, procalcitonin, C reactive protein, cytokines), organ function indices (cardiac, respiratory, hepatic, renal), coagulation variables (platelet counts, PT, INR, aPTT, fibrinogen, D-dimer, FDP, and antithrombin-III), and blood gas analyses will be recorded on the first, second, third, fifth and seventh days. Beyond that, treatment, including the volume of crystals, colloids, albumin and blood transfusion products, and urine volume, will be recorded every day. Outcomes, including ICU mortality, ventilator time, AEs/SAEs, and a follow-up visit on the 28th day, will be included.

All the data will be collected from the clinical electronic information system and recorded in the case report format (CRF). The data in the CRF recorded by researchers should be accurate, complete, and timely under the supervision and audit of two independent inspectors from the clinical research organization (CRO). All the CRFs will be recorded in an electronic document for statistical analysis. All participant centers were qualified by the National Medical Products Administration. The involved researchers complied with the Good Clinical Practice (GCP) guidelines during the study. All original documents will be stored in a locked site with restricted access at the Department of Critical Care Medicine of the First Affiliated Hospital of China Medical University for 10 years. The participant will be referred to by the participant code instead of the name in all the trial documents. Access will be granted to authorized person and the regulatory authorities to permit trial monitoring, audits, and reports.

### Data analysis

Data will be described as the mean and SD or median and IQR according to the data distribution. The frequency of ICU mortality per group with a risk difference and 95% CI and a corresponding OR and 95% CI will be reported. Kaplan–Meier analysis will be used to analyze the rate of death in a time-to-event analysis, and the survival difference will be tested using a Cox proportional hazards model. For univariate analysis, the difference will be compared between the UFH and placebo groups using the Mann–Whitney U test or 𝜒2 and Fisher’s exact test. A two-tailed *p* value <0.05 was considered statistically significant. All statistical analyses were conducted using SPSS 22.0 and GraphPad Prism 9.0 software (GraphPad Software, San Diego, CA). The register number, participants lost due to follow-up, and violations of the protocols will be reported. The missing data will be handled with multiple imputation method.

### Patient and public involvement

Patients and the public are not involved in the design of the study, including the development of research, outcome measures, enrollment, or conduct of the study. The results of the trial will be presented at conference or as a journal article. Authorship of any publication of results will include investigators and collaborators in the trial.

### Data safety monitoring committee

The independent data safety monitoring committee (DSMC) includes clinical practitioners, statistical specialists, legal and ethical professionals, and data management experts. Its responsibility is to oversee the conduct of the trial, to ensure the integrity of the design, and to protect the participants from avoidable risk. The clinical practitioners will be responsible for identifying potential recruiting and obtaining their consents. The DSMC will hold a meeting every three months to discuss about the process of the trial. The research will report to DSMC with the report of interim analysis and adverse events. The DSMC will undertake a review of enrolments and withdrawals in accordance with the DSMC Charter to ensure adequate study enrolment and the safety of participants. The DSMC will make suggestions to terminate, suspend, or modify the trial.

If the protocol requires amendments, it will be approved by the competent authority and the ethics committees. Protocol amendments will be updated on relevant clinical trial registries and sent to all the centers.

### Trial sponsor

The trial sponsor is the First Affiliated Hospital of China Medical University. The contact information is as follows: No.155, Nanjing North Street, Shenyang, China. Postcode: 110001. They ensure the provision of proper medical insurance compensation and bear certain legal liability before research begins. The sponsor has not participated in the study design; data collection management and analysis; interpretation of data; writing of the report, and the decision to submit the report for publication.

## Discussion

The trial is a multicenter, randomized, double-blinded RCT. We hypothesize that UFH will reduce ICU mortality compared with placebo in sepsis-induced DIC and has no obvious impact on bleeding.

The implication of anticoagulation and antithrombotic therapies for sepsis has drawn increasing attention during the last few years [23, 24]. Coagulation abnormalities are quite common in the early stage of sepsis. Endothelial injury, the formation of microthrombi, and excessive inflammatory factors act mutually and contribute to organ dysfunction or even death during sepsis [25]. However, some anticoagulants that only inhibit microthrombosis or replenish the loss of intrinsic anticoagulant factors have been proven to not improve organ function or reverse mortality in sepsis or septic shock [26]. Of note, the large RCTs on rhAPC allowed the use of heparin for preventing deep venous thrombosis and showed a survival benefit in the subgroups treated with heparin relative to the groups who did not receive heparin [9]. Intriguingly, the re-analysis of the three trials (the PROWESS using rhAPC [9], KyberSept using AT [10], OPTIMIST using rTFPI [27]) demonstrated that in the control group, the use of heparin can independently reduce 28-day mortality relative to the patients without heparin [28]. Furthermore, a single-center prospective randomized double-blind study (HETRASE) demonstrated that UFH had no beneficial effect on the survival of sepsis patients, although there was no increased risk of bleeding. However, the participants enrolled in the HETRASE study suffered from less severe sepsis with APAHCEII<10 and were more heterogeneous in the population [21].

In the trial, we enrolled participants with more rigorous criteria. The modified criteria, including platelet counts, prothrombin time, and D-dimer, are applied to diagnose DIC in septic patients. We exclude fibrinogen in the modified DIC criteria. The characteristics of DIC differ according to the underlying disease [23]. For trauma-induced DIC, excess fibrinolysis exists to counter massive thrombotic events, which shows decreased levels of fibrinogen [29]. In sepsis, coagulation activation occurs with injured fibrinolysis [23]. The imbalance of coagulation and fibrinolysis results in massive clot formation. Therefore, the level of fibrinogen is normal or even elevated in sepsis-induced DIC patients.

Heparin, a naturally occurring proteoglycan, is used in the prevention and treatment of venous thromboembolism due to its property of anticoagulation. Of note, heparin exerts anticoagulation effects dependent on AT levels. In addition to anticoagulation, heparin possesses anti-inflammatory effects. Our previous studies have demonstrated that heparin inhibits induced-lung inflammation by downregulating nuclear factor-kB and inhibits neutrophil recruitment in LPS-induced endotoxemia [15, 30]. Furthermore, UFH has been demonstrated to protect the endothelial glycocalyx via heparanase [31, 32] and ameliorate pulmonary microvascular endothelial dysfunction [33], which contributes to the improvement of microcirculation. Recently, Lu et al. showed that heparin inhibits thrombin formation, platelet aggregation, and fibrin aggregation in liver microvessels in an LPS model [34]. Mechanistically, heparin prevents caspase-11-dependent septic lethality independent of anticoagulant properties [35]^.^ Thus, we assume that UFH could prevent the development of DIC, ameliorate inflammatory responses, and improve microcirculation, which results in decreasing ICU mortality in septic patients.

Heparin or heparinoids are not routinely used for the prophylaxis of venous thromboembolism if patients have no high risk of thrombosis in China. Therefore, it is feasible to justify the efficacy of heparin in patients with sepsis-induced DIC in China. However, there are two main concerns about heparin administration in patients. One concern is the risk of major hemorrhage during the use of unfractionated heparin. Nonetheless, according to a previous clinical trial and the results of a meta-analysis, UFH is a safe intervention when it is used for sepsis [18, 21]. In the trial, UFH exerted anti-inflammatory and immunoregulatory effects at a dose of 100 U/kg/24 h, which is a relatively low dose. The other is the occurrence of HIT. The coagulation index and bleeding signs will be closely and consistently monitored during the entire study.

## Trial status

The trial opened to recruitment on 12 April 2018. The current protocol version is 1.3 (dated April 2017). Recruitment is expected to be completed by 31 December 2024.

### Supplementary Information

**Additional file 1.** SPIRIT Checklist for Trials.

**Additional file 2: Supplemental Figure 2.** Participanting Centres.

**Additional file 3.**

## Data Availability

The data produced by the trial will be confidential by the investigators, except to the extent that it is included in a publication as agreed in the publication policy of the protocol. Individual requests for access to the final dataset will be considered in discussion with the research ethics committee.
