# Supplementary material for: Efficacy and safety of heparin for sepsis-induced disseminated intravascular coagulation (HepSIC): study protocol for a multicenter randomized controlled trial
Source: Trials. 2024 Jan 2;25:4. doi: 10.1186/s13063-023-07853-5 (PMC10759642; doi:10.1186/s13063-023-07853-5)
Supplement: Supplementary file 2 — Additional file 2: Supplemental Figure 2. Participanting Centres. [file 13063_2023_7853_MOESM2_ESM.docx]

Supplemental Figure 2. Participanting Centres

| No. | Hospital | Province |
| --- | --- | --- |
| 1 | The First Afiiliated Hospital of China Medical University | Liaoning |
| 2 | The First Hospital of Jilin Univeristy | Jilin |
| 3 | Qilu Hospital of Shandong University（Qingdao) | Shandong |
| 4 | The Second Affiliated Hospital of Kunming Medical University | Yunnan |
| 5 | The First Affiliated Hospital of Dalian Medical University | Liaoning |
| 6 | The First Hospital of Qinhuangdao | Hebei |
| 7 | Zhongda Hospital of Southeast University | Jiangsu |
| 8 | The Second Affiliated Hospital of Dalian Medical University | Liaoning |
| 9 | First Affiliated Hospital of Harbin Medical University | Heilongjiang |
| 10 | Peking University People's Hospital | Beijing |
| 11 | The People's Hospital of Liaoning Province | Liaoning |
| 12 | West China Hospital of Sichuan Univerity | Sichuan |
| 13 | Fourth People's Hospital of Shenyang | Liaoning |
| 14 | Beijing Tsinghua Changgung Hospital | Beijing |
| 15 | The First Affiliated Hospital of Kunming Medical University | Yunnan |
| 16 | The Shengjing Hospital of China Medical Univeristy | Liaoning |
| 17 | The Affiliated Hospital of Qingdao University | Shandong |
| 18 | Beijing Friendship Hospital | Beijing |
| 19 | General Hospital of Ningxia Medical University | Ningxia |
| 20 | The First Affiliated Hospital of Nanchang University | Jiangxi |
