# Supplementary material for: Efficacy and safety of heparin for sepsis-induced disseminated intravascular coagulation (HepSIC): study protocol for a multicenter randomized controlled trial
Source: Trials. 2024 Jan 2;25:4. doi: 10.1186/s13063-023-07853-5 (PMC10759642; doi:10.1186/s13063-023-07853-5)
Supplement: Supplementary file 3 — Additional file 3. [file 13063_2023_7853_MOESM3_ESM.docx]

## Medical Research Ethics Committee of the First Affiliated Hospital of China Medical University

## Ethics review report form (annual, follow-up review)

Report date: September 6, 2023 (time of submission to the Ethics Committee )

| Ethics committee approval number | | | Kelun Trial [ 2019] No. 2015-1-5 | | | | Approval date | | December 30 , 2019 _ | | |
| --- | --- | --- | --- | --- | --- | --- | --- | --- | --- | --- | --- |
| project name | | | Standardized diagnosis and anticoagulation treatment standards for severe sepsis/septic shock-related coagulopathy  The establishment of the station | | | | | | | | |
| Sponsor/project source | | | Liaoning Provincial Health and Family Planning Commission/Liaoning Provincial Hospital Reform Key Clinical Department Diagnosis and Treatment Capacity Building Project | | | | | | | | |
| CRO | | | none | | | | | | | | |
| Responsible for the department | | | Department of Critical Care Medicine | | | | main researcher | | Ma Xiaochun | | |
| Team leader unit | | | The First Affiliated Hospital of China Medical University | | | | | | | | |
| Planned completion time | | | December 2024 | | | | | | | | |
| **Research progress** | **Subject information** | | | | | | | | | | |
|  | Total number of contract research cases | | | | 600 | | Number of enrolled cases | | | | 386 |
|  | Number of completed observation cases | | | | 368 | | Number of early exit cases | | | | 18 |
|  | Number of serious adverse events | | | | 0 | | Number of unexpected serious adverse events | | | | 0 |
|  | Number of serious adverse events reported | | | | 0 | | | | | | |
|  | **Research progress** | | | | | | | | | | |
|  | **research stage** | □Not started yet□Recruiting (not yet enrolled in the group)  ✔️The study is being implemented □The intervention of the subjects has been completed□Post-data processing | | | | | | | | | |
|  | Investigate whether risks exceed expectations | | | | | | | ✔️No □Yes (please attach a page for explanation) | | | |
|  | Any new information or developments that affect the risks and benefits of the research | | | | | | | ✔️No □Yes (please attach a page for explanation) | | | |
|  | There is an impact on the rights of subjects | | | | | | | ✔️No □Yes (please attach a page for explanation) | | | |
|  | Any researcher who may have a conflict of interest with this study | | | | | | | ✔️No □Yes (please attach a page for explanation) | | | |
|  | Participation or reduction in any collaborative research institution | | | | | | | □No✔️Yes ( please attach a page for explanation) | | | |
|  | Change researcher | | | | | | | ✔️No □Yes (please attach a page for explanation) | | | |
|  | Timely reporting of serious adverse events or important medical events that must be reported according to the protocol | | | | | | | □No□Yes✔️Not involved | | | |
| **Enrollment status of each center** | If the project is led by our center, fill in this column.  30 cases in the First Hospital of Jilin University , 24 cases in the Second Affiliated Hospital of Kunming Medical University , 31 cases in the Qingdao Branch of Qilu Hospital of Shandong University , 16 cases in the First Affiliated Hospital of Dalian Medical University , 15 cases in the First Affiliated Hospital of Qinhuangdao , Southeast 7 cases in University Zhongda Hospital, 7 cases in the Second Affiliated Hospital of Dalian Medical University, 10 cases in the First Affiliated Hospital of Harbin Medical University, 8 cases in Peking University People's Hospital, 4 cases in Liaoning Provincial People's Hospital, and 1 case in Shengjing Hospital Affiliated to China Medical University Examples include 1 case in Shenyang Fourth People's Hospital, 3 cases in West China Hospital of Sichuan University, 1 case in Tsinghua University Chang Gung Memorial Hospital, and 2 cases in the First Affiliated Hospital of Kunming Medical University | | | | | | | | | | |
| Is **it a research progress report submitted to the team leader’s unit** ? No✔️Yes | | | | | | | | | | | |
| Signature of the principal investigator | | | |  | | date | | | |  | |
| **Review method (the following content shall be filled in by the ethics committee)** | | | | | | | | | | | |
| Suggested review methods  □Meeting review  □Quick review  □Emergency meeting review | | | | | | | | | | | |
| Recipient’s signature: Date: | | | | | | | | | | | |

**Title: Ethical review opinions /approval documents** **No.: AF** **-** **SOP** **-** **07** **-** **1** **.** **2** **-** **01**

**Medical Research Ethics Committee of the First Affiliated Hospital of China Medical University**

**Ethical review opinions**

Kelun Trial【2023】No. 2015-1-6

| project name | | Establishment of a standardized platform for the standardized diagnosis and anticoagulation treatment of severe sepsis/septic shock-related coagulation disorders | | | | |
| --- | --- | --- | --- | --- | --- | --- |
| Sponsor/project source | | Liaoning Provincial Health and Family Planning Commission/Liaoning Provincial Hospital Reform Key Clinical Department Diagnosis and Treatment Capacity Building Project | | | | |
| CRO | | none | | | | |
| Team leader unit | | The First Affiliated Hospital of China Medical University | | | | |
| Responsible for the department | | Department of Critical Care Medicine | | main researcher | | Ma Xiaochun |
| review category | | Annual, follow-up review | | review method | | meeting review |
| Review materials list | | | | | | |
| ■ Ethics review application form (annual, follow-up review)  ■ Add description of participating units | | | | | | |
| Meeting status | | | | | | |
| Number of people attending the meeting: 10 people | | | | Abstain or avoid: 0 people | | |
| approve | Approved after modification | | Review again after modification | | not approved | terminate/pause |
| 10 votes | 0 votes | | 0 votes | | 0 votes | 0 votes |
| 1. The ethics committee’s review decision on the project is as follows ( mark × within □ )  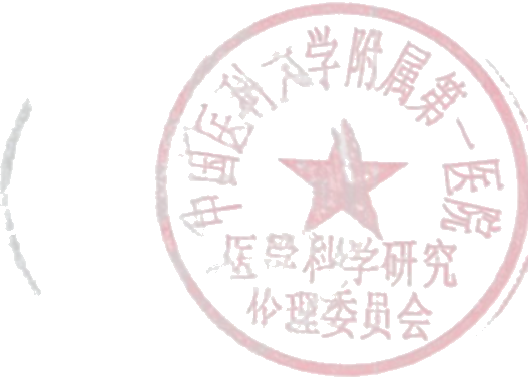🗵 Approve □Approve after modification □ Review again after modification □Disapproval □Terminate/suspend  2. After review by the Ethics Committee, it is deemed that the annual and follow-up reports meet the requirements.  3. This ethical review opinion does not serve as the basis for submission to the State Food and Drug Administration for drug/device registration .  Signature of the Director (Deputy Director) :  Ethics Committee Court:  Date : | | | | | | |
| Remark:  1. The Ethics Committee has decided that the frequency of follow-up review of this project is 12 months. Please submit the annual and follow-up review report one month in advance according to the review frequency;  2. The applicant is requested to submit an annual and follow-up review report in accordance with the ethics committee’s previous review frequency for the project, and then submit an annual and follow-up review report in accordance with the new review frequency and requirements ;  3. Correspondence address of the Ethics Committee: No. 155, Nanjing North Street, Heping District, Shenyang City, Liaoning Province, Postal Code: 110001, Office Telephone  024-83282837. | | | | | | |
